# Medical History from the Earliest Times

**Published:** 1892-10-08

**Authors:** E. T. Withington


### Medical History from the Earliest Times.

By E. T. Withington, M.A., M.B. Oxon.

### XXVI.—THE ECLECTICS AND COMPILERS.

Eclecticism is a term attractive to the cultured mind, for it implies the essence of culture, the choice of what is best from whatever source it comes. But if the sanguine student of medical history, hearing of the rise of a school of eclectic practitioners, should fancy he has at last arrived at a golden age, when sects and systems vanished, and great and liberal-minded physicians carried the healing art to heights of excellence unknown before, he would be grievously disappointed. Whatever eclectics may have done for other departments of knowledge they did little for the progress of medicine. The title has, indeed, been applied to the greatest physicians, Hippocrates, Heraclides, Galen, Soranus, and to our own modern medicine; and, in some sense, it may be claimed by all who are not absolutely blinded by the fanaticism of their sect or system; but the medical eclectics, strictly so-called, formed a distinct school which had many sub-divisions. Some, like our old acquaintance Archigenes, formed systems of their own, and these were the best, they had at least enthusiasm; but the liberal-mindedness of others was the result of that baser scepticism which despairs of progress, and has no wish to overcome itself, or of the ignorance which seeks a cheap reputation for knowledge by pretending to have weighed everything and found it wanting. Between these extremes the eclectic school comprised many able and excellent physicians, though most of them show an unfortunate tendency either to dilettantism or compilation. First, both in age and rank, is Aretæus of Cappadocia (A.D. 50 130 (?) ), a scholarly physican who lived apart from the controversies of his time, and composed in the old Ionic dialect word pictures of disease, the truth and vigour of which are universally recognised. But his writings, excellent as they are, had little influence on medical history, and as they are readily accessible to English readers, need not be further considered here. What the physicians of that age required was, not a reversion to Hippocrates and Nature, but to be told on good authority what they were to think, and above all what remedies they were to employ in each separate disease; and the typical products of the post-Galenic epoch are a series of compilations, varying in size and value, but having certain tendencies in common. We first meet with large compilations, which gradually supplant the works of original writers, and sometimes cause their complete disappearance. These are replaced in their turn by shorter abstracts and epitomes, in which the therapeutic part is enlarged at the expense of the other divisions of medical science, till we finally get to works containing merely the names of diseases, followed by long lists of drugs which will more or less certainly cure them. At the revival of science we shall be able to trace a process ex`ctly the reverse of this, and shall find the mediæval receipt-books gradually replaced by the short compendia of the Salernitan masters, which themselves disappear before the great Arabic compilations, the "Continens" of Rhazes, and the "Canon" of Avicenna, till at last we arrive once more at original writings. At the same time, the study of the nature, diagnosis, and causes of disease re-assumes its proper position, and in our modern medical handbooks we can point triumphantly to pages of pathology followed by a paragraph on treatment, a proportion which, curious as it may seem, is as much the sign of a progressive as the reverse is the mark of a decadent age in medicine.

The first and greatest of the compilers was Oribasius of Pergamus, physician and friend of the Emperor Julian (360-363), at whose suggestion he composed the seventy books of his "Medical Collections." Only about a third of this work survives, but it is of great historical value, for Oribasius invariably mentions his authorities, and quotes them with exactness, and it is to him that we owe most of our knowledge of such practitioners as Antyllus and Archigenes.

Oribasius is the last of the great pagan physicians, but the first important Christian writer on medicine lived nearly two centuries later. This was Aëtius of Amida, who held the title of Count (*comes obsequii*) at the Byzantine Court, probably under Justinian I. (527-565), and composed the second great medical compilation, the Tetra-biblos, in sixteen books. Amida on the Tigris was one of the most easterly outposts of Greek civilisation, and it is interesting to notice that Aëtius makes the earliest mention of such Eastern drugs as cloves and camphor, which were afterwards more fully introduced into medicine by the Arabs. His work is especially distinguished by its long lists of complicated prescriptions, and the passages which indicate his religion are of a somewhat ominous character. Thus, if a patient has a bone in his throat, it may be extracted by forceps, or he may be given a piece of raw meat on a string, to be pulled up when he has swallowed it, or thus "Bid the patient attend to you, and say, 'Bone (or whatever it is) come forth, like as Christ brought Lazarus from the tomb and Jonah from the whale.' Then take him by the throat, and say, 'Blasius, martyr and servant of Christ, saith Either come up or go down.'" Elsewhere, in describing an ointment, he declares that it is necessary to repeat continually during its preparation, "The God of Abraham, Isaac, and Jacob give efficacy to this salve."

About a century after Aëtius a third medical epitome was complied, in seven books, by Paul of Ægina, the last important product of the great school of Alexandria. Aëtius and Paul resemble one another in many respects; both are largely indebted to Galen and Oribasius; both seem to have added some original matter, but as neither hesitates to quote his predecessors, even verbally, without mentioning their names, it is impossible to decide how far this originality is genuine. While Aëtius devotes himself chiefly to medicine, the most brilliant part of Paul's epitome is the sixth, or surgical book, which was much valued by the Arabs, who estimated still more highly his work on diseases of women (now lost), and gave him the honourable title of the "Obstetrician."

Among the writers whom Paul frequently quotes, but rarely mentions is Alexander of Tralles, a younger contemporary of Aëtius, an eclectic of the highest type, who may be considered the last of the great

Greek physicians, as his brother Anthemius, builder of the church of St. Sophia, is the last of the great Greek architects. His pathology, indeed, is taken entirely from Galen, whom he never mentions without the epithet "most divine," but in questions of treatment he often contradicts his master's directions when opposed to his own experience. While adopting many of the excellent general rules of the Methodists, he insists, in truly Hippocratic language, on the necessity of considering the individual patient, and on the folly of treating diseases by a rule of thumb; and though his works show the usual tendency to long lists of prescriptions, including some of a very curious nature, he never omits to assert that careful diagnosis should precede treatment, and to point out the importance of discovering and attacking the cause of the disorder.

The stream of Greek medicine, which we have followed for more than a thousand years, now divides into three branches, each of which must be traced separately. The first and most direct continuation flows in a narrow and sluggish current, whose bed is sometimes nearly choked by the rocks of theological controversy, and ends abruptly amid the ruins of the Eastern Empire. The second, for a time the broadest and clearest of the three, turns eastward through fresh fields; upon its banks grow healing plants unknown before—senna, cubebs, orange, and tamarind—and in its waters—if we may continue the metaphor—swim strange fish, the chemist and the apothecary, till finally bending to the west, it empties itself almost entirely into another stream. This third or western branch, rising in the marshes of ignorance, and covered by the mists of superstition, gradually grows wider and more rapid, and receiving copious affluents from the other two, finally develops into a mighty river flowing "with pomp of waters unwithstood," and bearing in its bosom, let us hope, the healing of the nations.

